# Left-Right Differences in Oral Function and Quality of Life of Patients Who Underwent Tongue Resection

**DOI:** 10.7759/cureus.71831

**Published:** 2024-10-19

**Authors:** Yuka Harada, Yoshiaki Ihara, Tomoki Tamai, Mitsunori Ishiguro, Yuichi Tashimo, Shinji Nozue, Kouta Nagoya, Toshikazu Shimane

**Affiliations:** 1 Division of Oral Rehabilitation Medicine, Department of Oral Health Management, School of Dentistry, Showa University, Tokyo, JPN; 2 Division of Oral Functional Rehabilitation Medicine, Department of Oral Health Management, School of Dentistry, Showa University, Tokyo, JPN; 3 Department of Oral Functional Rehabilitation Medicine, Showa University Graduate School of Dentistry, Showa University, Tokyo, JPN; 4 Department of Otorhinolaryngology Head and Neck Surgery, School of Medicine, Showa University, Tokyo, JPN

**Keywords:** feeding, oral function, quality of life (qol), tongue cancer, tongue resection

## Abstract

Background

The tongue plays an important role in oral functions, including articulation, eating, and swallowing. The treatment of tongue cancer (TC) often leads to oral morbidities, such as limited mouth opening, dry mouth, swallowing difficulties, and mucositis. The anterior-posterior resection area of the tongue has a significant impact on oral function and quality of life (QOL). However, the specific left-right differences in oral function of patients who underwent tongue resection remain unclear. This study aimed to investigate the effect of left-right differences on oral function and QOL in patients with TC who underwent tongue resection.

Methodology

This study included 40 patients with TC who underwent surgical resection smaller than hemiglossectomy and were divided into left (LG) and right (RG) tongue groups. The oral, respiratory, and feeding functions and the QOL at baseline (before treatment) and one, three, and six months after treatment (1M, 3M, and 6M, respectively) were evaluated. Maximum tongue pressure and lip-closure force were used to evaluate the oral functions. Peak expiratory flow was used to evaluate the respiratory function. The Functional Oral Intake Scale was used to evaluate the feeding functions. The QOL was assessed using the European Organization for Research and Treatment of Cancer (EORTC) QOL Questionnaires: QLQ-C30, QLQ-H&N35.

Results

The results indicated that there were no significant differences in oral, respiratory, or feeding function between the LG and RG groups at all evaluation periods. However, regarding QOL assessments, at 3M, LG indicated a significantly higher score than RG for swallowing (LG = 3.17, RG = 10.00, p = 0.037) and trouble with social eating (LG = 12.30, RG = 20.56, p = 0.025) in the EORTC QLQ-H&N35. At 6M, the LG also indicated a significantly higher QOL than RG for global health status (LG = 81.14, RG = 65.69, p = 0.018) and physical function (LG = 96.51, RG = 89.80, p = 0.047) in the EORTC QLQ-C30.

Conclusions

While the left-right differences in tongue function did not lead to functional impairment post-surgery, they significantly affected the QOL. The results indicate the importance of considering these differences in postoperative care to effectively address the QOL concerns.

## Introduction

Head and neck cancer is the 15th most common type of cancer in Japan [1]. Tongue cancer (TC) is the most frequent type of head and neck cancer, accounting for approximately 25% of all head and neck cancers [2]. It has been reported that the rate of new TC cases was 3.6 per 100,000 men and women per year, and the rate of new TC cases increased by an average of 2.2% each year from 2010 to 2019 [3]. The treatment of head and neck cancers often causes various complications, and patients with head and neck cancers complain of oral complications caused by radiation, such as limited mouth opening (trismus), dry mouth (xerostomia), swallowing problems (dysphagia), and mucositis. In particular, the treatment of TC leads to several oral dysfunctions, including difficulty in swallowing and speech [4,5]. Previous studies have reported that the area of the tongue resected due to TC has a significant effect on swallowing and speech [6]. In particular, dysphagia has been reported in more than 40% of patients with TC who underwent tongue resection [7,8]. The resected area of the tongue has been reported to have a significant effect on patients’ oral function and quality of life (QOL) [9,10]. However, the left-right differences in oral function due to the resected area of the tongue remain unclear. In terms of limb function, there is a dominant side such as the dominant hand or leg. Oral function has been reported to be non-uniform between the left and right sides. For example, laterality of stomatognathic function and habitual mastication have been reported [11]. In addition, lateral misarticulation in the use of the tongue between the left and right sides was observed even in the absence of organic abnormalities [12]. However, there are no reports on the left-right differences in the use of the tongue in swallowing. Thus, this study aimed to investigate the differences in tongue function and its effect on patients’ QOL after surgical treatment of patients with TC who underwent tongue resection smaller than hemiglossectomy.

This article was previously posted to the medRxiv preprint server on May 2, 2024.

## Materials and methods

Patients

This study included 40 patients with TC who were scheduled for surgical resection smaller than hemiglossectomy (partial glossectomy or hemiglossectomy) at the Head and Neck Oncology Center, Showa University Hospital, and were subsequently referred to the Department of Special Needs Dentistry, Division of Oral Rehabilitation Medicine, Showa University Dental Hospital for rehabilitation. The participants were divided into the following two groups: those with TC on the left (LG) and right (RG) sides.

The exclusion criteria were as follows: age <20 years, inability to follow instructions, presence of other malignant tumors, and effect of other diseases on oral function. All measurements were performed at baseline (BL) and one (1M), three (3M), and six (6M) months after treatment.

This study was approved by the Ethics Committee of Showa University School of Medicine (approval number: 2355), and all participants received both oral and written instructions and signed an approved informed consent form before participating in the study. All procedures were performed in accordance with the World Medical Association Declaration of Helsinki (version 2002).

Assessments

All evaluations were performed by dentists from the Division of Oral Functional Rehabilitation Medicine, Department of Oral Health Management, School of Dentistry, Showa University, Japan. Data regarding age, sex, primary TC side, TMN classification, surgical method, and medical history were collected from the medical records. Maximum tongue pressure (MTP) and lip-closure force (LCF) were used to evaluate oral functions. Peak expiratory flow (PEF) was used to evaluate respiratory function. The Functional Oral Intake Scale (FOIS) was used to evaluate feeding function. The patients’ QOL was assessed using the European Organization for Research and Treatment of Cancer (EORTC) QOL Questionnaires: QLQ-C30 and QLQ-H&N 35.

Oral function

The MTP was measured using a JMS tongue pressure measurement device (JMS Co., Ltd., Hiroshima, Japan). A tongue pressure probe with a balloon at its tip was placed behind the upper front teeth. The participants were instructed to press the balloon against their palate. The participants pressed the balloon for five seconds, and the maximum pressure inside the balloon was measured. The measurements were performed 10 times, and the average value was calculated as the participant’s MTP score [13]. There was an interval of at least one minute to recover from fatigue.

The LCF was measured using a lip-force measuring device (Lip de Cum model LDC-110R, Cosmo-Instruments Co., Ltd., Tokyo, Japan). The lip pads of the instruments were inserted between the upper and lower lips. The participants were instructed to close their upper and lower lips, and the maximum LCF was recorded. The measurements were performed five times, and the average value was calculated as the participant’s LCF score [14]. There was an interval of at least one minute to recover from fatigue.

Respiratory function

PEF was measured using a peak flow meter (Mini Wright®, Clement Clarke International Ltd., UK). The participants were instructed to take a deep breath, fill their lungs completely, place the mouthpiece in their mouth, and blast the air out as hard and as fast as possible with a single blow. Subsequently, the number of peak flows was determined. The measurements were performed five times, and the average value was calculated as the participant’s PEF score.

Feeding function

The FOIS was used to measure the participants’ feeding (eating) status [15]. The FOIS is a valid and reliable tool for documenting functional eating abilities. A seven-point ordinal scale was used to describe the functional oral intake of patients with dysphagia (from 0: nothing by month to 7: total oral diet with no restrictions).

QOL measurements

The participants’ QOL was assessed using the EORTC QLQ-C30 version 3.0 and QLQ-H&N35 questionnaires, the Japanese version. The scores were calculated according to the EORTC scoring manual [16]. The EORTC QLQ-C30 consisted of both single- and multiple-item scales and included a global health status/QOL, five functional, three symptom, and six single-item scales. Each multi-item scale contained different items and no item appeared on more than one scale. The scores for all scales and single-item scales ranged from 0 to 100. Higher scores indicated higher response levels. For the functional scale, a high score represented a high/healthy level of functioning, and a high score for the global health status/QOL represented a high QOL. However, a high score on the symptom scale/item represented a greater number of symptoms or problems. The EORTC QLQ-H&N35 included pain, swallowing, sensation (taste and smell), language, social diet, social contact, and sexuality, and incorporated seven multi-item scales that assessed social contact and sexuality. Eleven individual items were included in this study. All scales and single items ranged from 0 to 100. Higher scores indicated more problems for all items and scales.

Rehabilitation

All participants in this study received indirect or direct training, as needed. After the pain in the surgical region subsided, the participants underwent rehabilitation such as massage for surgical scarring, oral motor exercises including lingual exercises, and direct training using various food materials as needed. Rehabilitation was performed by a dentist or speech-language pathologist.

Statistical analysis

Bivariate analyses with t-tests, chi-square test, and Fisher’s exact test were used to compare the two groups for age, gender, oral function measurements, feeding function, and QOL measurements. All statistical analyses were performed using SPSS version 25.0 (IBM Corp., Armonk, NY, USA). All p-values were two-sided, and p-values lower than 0.05 were considered significant.

## Results

Participants

A total of 40 patients (25 men, and 15 women) participated in this study. The mean age was 59.93 years (standard deviation (SD) = 14.50 years). Based on the primary side of TC, 23 and 17 participants were classified as LG and RG, respectively. The LG included 13 participants with T1N0M0, six with T1N1M0, six with T2N0M0, two with T2N0M0, and two with T3N0M0. The RG included eight participants with T1N0M0, seven with T2N0M0, one with T3N0M0, and one with T3N1M0. Regarding the surgical methods of treatment for TC, the LG group included 19 and four participants who underwent partial glossectomy and hemiglossectomy, respectively. The RG included 15 and two participants who underwent partial glossectomy and hemiglossectomy, respectively. The characteristics of each group are presented in Table 1.

**Table 1:** Participant characteristics. LG: left group; RG: right group; TC: tongue cancer

	TC on the left side of the tongue group (LG)	TC on the right side of the tongue group (RG)
Gender (male:female)	14:9	11:6
Age in years, mean (SD)	56.87 (14.83)	64.06 (13.36)
Methods of surgical treatment		
Partial glossectomy: hemiglossectomy	19:4	15:2
TNM classification		
T1N0M0	13	8
T1N1M0	1	0
T2N0M0	6	7
T2N1M0	1	0
T3N0M0	2	1
T3N1M0	0	1

Oral function

MTP

**Figure 1:**
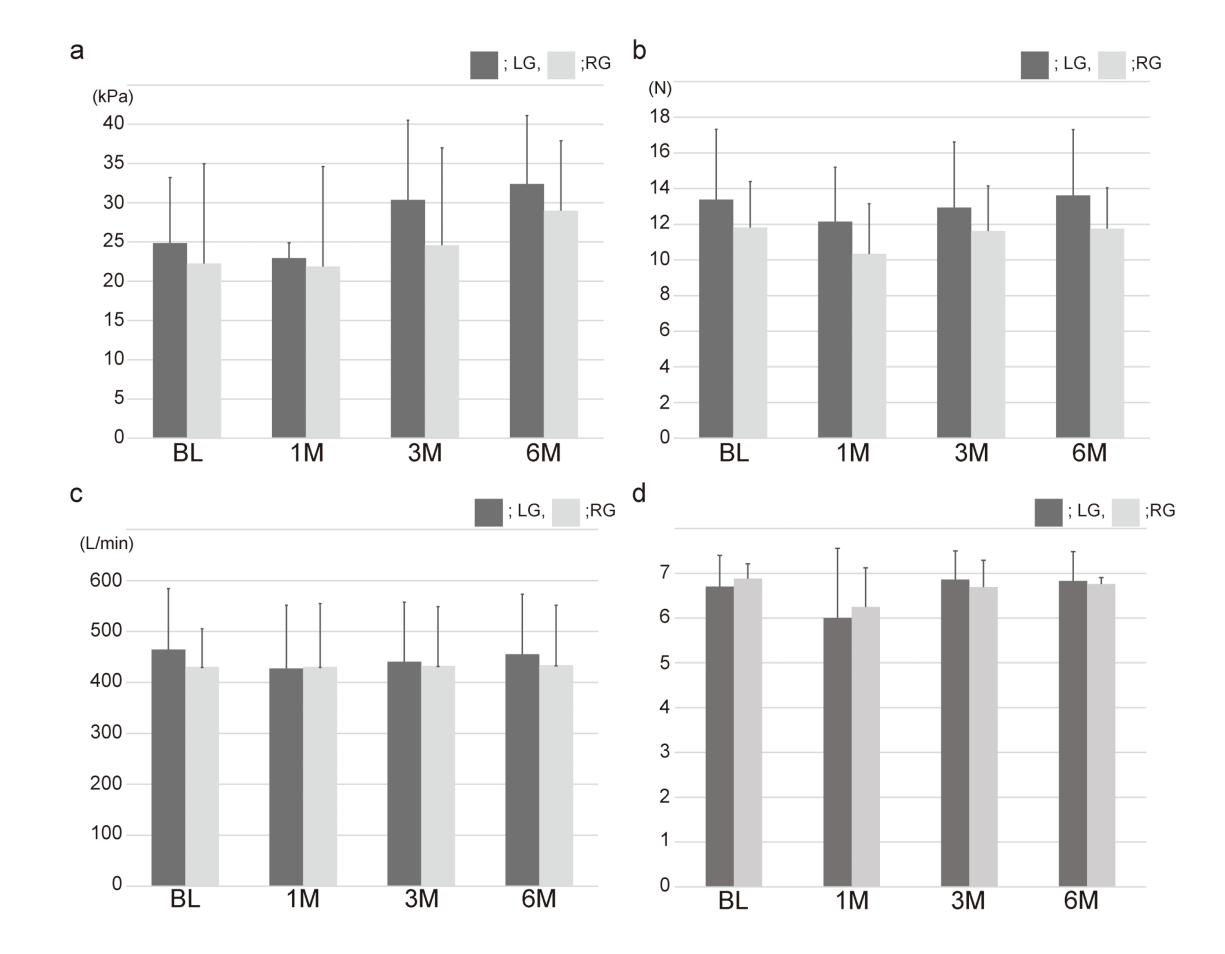
The results of the measurements. a: Results of maximum tongue pressure. b: Results of lip closure force. c: Results of peak expiratory flow. d: Results of the functional oral intake scale. There was no significant difference between the groups in all measurements. LG: left group; RG: right group; BL: baseline; M: month

At BL, the mean MTP of LG was 24.87 kPa (SD = 8.33) and that of RG was 22.27 kPa (SD = 12.72). At 1M, the mean MTP of LG was 22.93 kPa (SD = 1.98) and that of RG was 21.78 kPa (SD = 12.73). At 3M, the mean MTP of LG was 30.38 kPa (SD = 10.13) and that of RG was 24.57 kPa (SD = 12.44). At 6M, the mean MTP of LG was 32.41 kPa (SD = 8.68) and that of RG was 29.00 kPa (SD = 8.89). There were no significant differences between LG and RG at any evaluation period (BL, p = 0.538; 1M, p = 0.760; 3M, p = 0.139; 6M, p = 0.346) (Figure 1, Panel a).

LCF

At BL, the mean LCF of LG was 13.38 N (SD = 3.94) and that of RG was 11.83 N (SD = 2.56). At 1M, the mean LCF of LG was 12.15 N (SD = 3.05) and that of RG was 10.34 N (SD = 2.82). At 3M, the mean LCF of LG was 12.94 N (SD = 3.67) and that of RG was 11.62 N (SD = 2.53). At 6M, the mean LCF of LG was 13.62 N (SD = 3.69) and that of RG was 11.76 N (SD = 2.28). There were no significant differences between LG and RG at any evaluation period (BL, p = 0.186, 1M; p = 0.072, 3M; p = 0.203, 6M; p = 0.074) (Figure 1, Panel b).

Respiratory function

At BL, the mean PEF of LG was 464.33 L/minute (SD = 120.07) and that of RG was 430.46 L/minute (SD = 74.52). At 1M, the mean PEF of LG was 427.30 L/minute (SD = 124.12) and that of the RG was 430.43 L/minute (SD = 66.21). At 3M, the mean PEF of LG was 440.76 L/minute (SD = 116.61) and that of RG was 432.27 L/minute (SD = 76.94). At 6M, the mean PEF of LG was 455.63 L/minute (SD = 117.62) and that of RG was 433.71 L/minute (SD = 102.96). There were no significant differences between LG and RG at any evaluation period (BL, p = 0.342, 1M; p = 0.921, 3M; p = 0.794, 6M; p = 0.565) (Figure 1, Panel c).

Feeding function

At BL, the mean FOIS score of LG was 6.70 (SD = 0.70) and that of RG was 6.88 (SD = 0.33). At 1M, the mean FOIS score of LG was 6.00 (SD = 1.56) and that of RG was 6.25 (SD = 0.87). At 3M, the mean FOIS score of LG was 6.86 (SD = 0.64) and that of RG was 6.69 (SD = 0.60). At 6M, the mean FOIS score of LG was 6.83 (SD = 0.65) and that of RG was 6.76 (SD = 0.14). There were no significant differences between LG and RG at any evaluation period (BL, p = 0.272, 1M; p = 0.564, 3M; p = 0.392, 6M; p = 0.751) (Figure 1, Panel d).

QOL measurements

EORTC QLQ-C30

Table 2 presents the results of the EORTC QLQ C-30. At BL, there were no significant differences between LG and RG (global health status, p = 0.422; physical functioning, p = 0.610; role functioning, p = 0.273; emotional functioning, p = 0.340; cognitive functioning, p = 0.424; social functioning, p = 0.289; fatigue, p = 0.726; nausea and vomiting, p = 0.341; pain, p = 0.957; dyspnea, p = 0.334; sleep, p = 0.405; appetite loss, p = 0.832; constipation, p = 0.743; diarrhea, p = 0.912; financial difficulties; p = 0.200).

**Table 2:** Result of European Organization for Research and Treatment of Cancer Quality of Life Questionnaire-C30. LG: left group; RG: right group; BL: baseline; M: month

		BL	1M	3M	6M
		Mean (SD)	Mean (SD)	Mean (SD)	Mean (SD)
Global health status	LG	64.58 (24.25)	78.97 (18.56)	79.37 (16.38)	81.14 (18.81)*
	RG	71.15 (19.13)	68.89 (21.47)	77.22 (17.10)	65.69 (18.37)
Physical functioning	LG	98.67 (3.74)	94.92 (8.67)	97.14 (4.51)	96.51 (5.82)*
	RG	98.97 (3.70)	93.33 (9.06)	88.44 (16.23)	89.80 (12.05)
Role functioning	LG	98.89 (4.30)	88.10 (3.28)	96.03 (8.98)	96.83 (8.53)
	RG	89.74 (28.50)	88.10 (4.43)	87.78 (23.96)	96.08 (9.37)
Emotional functioning	LG	80.56 (16.86)	88.89 (13.52)	94.05 (9.18)	90.35 (12.50)
	RG	87.88 (6.09)	87.18 (5.94)	87.78 (21.33)	83.33 (24.40)
Cognitive functioning	LG	90.00 (15.17)	92.06 (13.56)	92.86 (11.27)	88.60 (15.77)
	RG	81.82 (9.12)	91.67 (12.66)	82.22 (27.79)	82.14 (32.33)
Social functioning	LG	92.22 (12.39)	90.48 (17.14)	98.41 (7.27)	97.37 (8.36)
	RG	81.82 (8.83)	85.71 (18.32)	97.78 (8.61)	91.67 (19.34)
Fatigue	LG	14.07 (14.22)	20.11 (22.67)	12.70 (15.43)	15.79 (14.49)
	RG	16.16 (15.21)	17.46 (17.82)	24.44 (28.54)	15.08 (22.48)
Nausea and vomiting	LG	0.00 (0)	1.59 (5.01)	0.79 (3.63)	3.17 (14.55)
	RG	1.52 (5.03)	1.19 (4.54)	0.00 (0.00)	0.00 (0.00)
Pain	LG	23.33 (25.04)	15.87 (17.85)	8.73 (14.55)	8.33 (14.29)
	RG	22.73 (30.07)	12.82 (16.88)	20.00 (32.24)	6.41 (16.01)
Dyspnea	LG	2.22 (8.61)	1.59 (7.27)	1.59 (7.27)	4.76 (11.95)
	RG	0.00 (0)	2.38 (8.91)	15.56 (9.12)	4.76 (17.82)
Sleep	LG	20.00 (24.56)	11.11 (16.10)	4.76 (11.95)	5.00 (16.31)
	RG	12.12 (6.78)	15.38 (22.01)	15.56 (24.77)	0.00 (0.00)
Appetite loss	LG	2.22 (8.61)	6.35 (17.06)	1.59 (7.27)	4.76 (21.82)
	RG	3.03 (10.05)	9.53 (4.18)	13.33 (35.19)	4.76 (17.82)
Constipation	LG	11.11 (24.12)	6.35 (13.41)	6.35 (17.06)	4.76 (11.95)
	RG	15.15 (34.52)	21.43 (30.96)	0.00 (0.00)	4.76 (12.10)
Diarrhea	LG	6.67 (13.80)	7.94 (17.97)	3.17 (10.03)	12.96 (25.92)
	RG	6.06 (13.48)	0.00 (0.00)	8.89 (15.26)	2.56 (9.25)
Financial difficulties	LG	11.11 (20.57)	11.11 (24.34)	4.76 (21.82)	7.41 (24.40)
	RG	3.03 (10.05)	7.69 (14.62)	6.67 (13.80)	0.00 (0.00)

At 1M, there was no significant difference between LG and RG (global health status, p = 0.154; physical functioning, p = 0.610; role functioning, p = 1.000; emotional functioning, p = 0.800; cognitive functioning, p = 0.930; social functioning, p = 0.446; fatigue, p = 0.703; nausea and vomiting, p = 0.808; pain, p = 0.620; dyspnea, p = 0.784; sleep, p = 0.551; appetite loss, p = 0.575; constipation, p = 0.105; diarrhea, p = 0.066; financial difficulties, p = 0.612).

At 3M, there was no significant difference between LG and RG (global health status, p = 0.709; physical functioning, p = 0.061; role functioning, p = 0.221; emotional functioning, p = 0.299; cognitive functioning, p = 0.179; social functioning, p = 0.818; fatigue, p = 0.163; nausea and vomiting, p = 0.329; pain, p = 0.222; dyspnea, p = 0.153; sleep, p = 0.135; appetite loss, p = 0.222; constipation, p = 0.104; diarrhea, p = 0.218; financial difficulties, p = 0.751).

At 6M, the mean global health status and physical functioning scores of LG were significantly higher than those of RG (global health status, p = 0.018; physical functioning, p = 0.047). Other scores were not significantly different between LG and RG (role functioning, p = 0.801; emotional functioning, p = 0.323; cognitive functioning, p = 0.500; social functioning, p = 0.316; fatigue, p = 0.919; nausea and vomiting, p = 0.329; pain, p = 0.733; dyspnea, p = 1.000; sleep, p = 0.186; appetite loss, p = 1.000; constipation, p = 1.000; diarrhea, p = 0.131; financial difficulties, p = 0.215).

EORTC QLQ-H&N35

Table 3 presents the results of EORTC QLQ H&N35. At BL, there was no significant difference between LG and RG (pain, p = 0.870; swallowing, p = 0.823; sense problem, p = 0.189; speech problems, p = 0.456; trouble with social eating, p = 0.671; trouble with social contact, p = 0.916; less sexuality, p = 0.726; teeth, p = 0.926; mouth opening, p = 0.072; dry mouth, p = 0.158; sticky saliva, p = 0.347; coughing, p = 0.892; feeling ill, p = 0.396; pain killers, p = 0.620; nutritional supplements, p = 0.082; feeding tube, p = 0.341; weight loss, p = 0.912; weight gain, p = 0.445).

**Table 3:** Result of European Organization for Research and Treatment of Cancer Quality of Life Questionnaire-H&N 35. LG: left group; RG: right group; BL: baseline; M: month

		BL	1M	3M	6M
		Mean (SD)	Mean (SD)	Mean (SD)	Mean (SD)
Pain	LG	19.44 (14.32)	11.51 (8.93)	5.95 (9.55)	4.58 (6.88)
	RG	18.18 (22.00)	8.33 (10.21)	5.00 (6.90)	2.99 (7.39)
Swallowing	LG	18.89 (23.46)	10.32 (14.65)	3.17 (7.67)*	3.33 (7.84)
	RG	16.67 (25.55)	14.10 (21.89)	10.00 (10.06)	4.49 (8.75)
Senses problems	LG	3.33 (9.34)	3.97 (8.98)	0.79 (3.64)	2.50 (6.11)
	RG	0.00 (0.00)	6.41 (12.80)	10.00 (23.40)	2.56 (6.26)
Speech problems	LG	15.56 (22.93)	16.40 (12.48)	6.88 (10.23)	6.67 (6.88)
	RG	9.09 (20.38)	25.64 (24.17)	17.78 (23.68)	8.12 (16.45)
Trouble with social eating	LG	22.78 (19.28)	25.40 (21.81)	12.30 (13.34)*	12.70 (10.41)
	RG	26.52 (23.52)	25.00 (21.25)	20.56 (7.63)	18.59 (12.80)
Trouble with social contact	LG	7.14 (12.11)	8.89 (12.88)	2.22 (6.09)	1.67 (4.78)
	RG	7.88 (19.96)	14.44 (15.26)	8.44 (14.79)	1.54 (3.99)
Less sexuality	LG	15.38 (23.04)	14.17 (25.52)	0.00 (0.00)	24.07 (30.90)
	RG	19.70 (34.01)	6.94 (16.60)	7.69 (14.62)	10.71 (18.03)
Teeth	LG	11.11 (20.57)	3.17 (14.55)	6.35 (17.06)	7.94 (20.83)
	RG	12.12 (30.81)	7.14 (14.19)	2.22 (8.61)	2.38 (8.91)
Opening mouth	LG	26.67 (25.82)	12.70 (22.30)	3.17 (10.03)	1.59 (7.27)
	RG	9.09 (21.56)	14.29 (17.12)	6.67 (13.80)	9.52 (20.37)
Dry mouth	LG	24.44 (32.04)	23.81 (28.17)	15.87 (27.12)	12.70 (16.59)
	RG	9.09 (21.56)	14.29 (21.54)	33.33 (37.80)	16.67 (21.68)
Sticky saliva	LG	26.67 (28.73)	15.87 (20.05)	6.35 (22.65)	7.94 (14.55)
	RG	15.15 (31.14)	16.67 (31.35)	20.00 (24.56)	14.29 (17.12)
Coughing	LG	11.11 (20.57)	9.52 (15.43)	6.35 (17.06)	7.94 (14.55)
	RG	12.12 (16.82)	7.14 (14.19)	8.89 (15.26)	2.38 (8.91)
Felt ill	LG	24.44 (26.63)	12.70 (19.65)	7.94 (23.34)	7.94 (14.55)
	RG	15.15 (27.34)	26.19 (29.75)	15.56 (24.77)	19.05 (17.12)
Pain killers	LG	8.89 (15.26)	7.94 (14.55)	3.17 (10.03)	1.59 (7.27)
	RG	12.12 (16.82)	11.90 (16.57)	2.22 (8.61)	0.00 (0.00)
Nutritional supplements	LG	6.67 (13.80)	3.17 (10.03)	4.76 (11.95)	6.34 (13.41)
	RG	0.00 (0.00)	4.76 (12.10)	0.00 (0.00)	2.38 (8.91)
Feeding tube	LG	0.00 (0.00)	0.00 (0.00)	0.00 (0.00)	0.00 (0.00)
	RG^f^	3.03 (10.05)	0.00 (0.00)	0.00 (0.00)	0.00 (0.00)
Weight loss	LG	6.67 (13.80)	6.35 (13.41)	3.17 (10.03)	3.17 (10.03)
	RG	6.06 (13.48)	7.69 (14.62)	6.67 (13.80)	2.38 (8.91)
Weight gain	LG	6.67 (13.80)	15.87 (17.06)	11.11 (16.10)	9.52 (15.43)
	RG	3.03 (10.05)	10.26 (16.01)	20.00 (16.90)	16.67 (17.30)

At 1M, there was no significant difference between LG and RG (pain, p = 0.365; swallowing, p = 0.588; senses problems, p = 0.554; speech problems, p = 0.220; trouble with social eating, p = 0.959; trouble with social contact, p = 0.301; less sexuality, p = 0.340; teeth, p = 0.429; mouth opening, p = 0.814; dry mouth, p = 0.267; sticky saliva, p = 0.934; coughing, p = 0.642; feeling ill, p = 0.151; pain killers, p = 0.473; nutritional supplements, p = 0.688; feeding tube, p = 1.000; weight loss, p = 0.791; weight gain, p = 0.341).

At 3M, the mean swallowing and trouble with social eating scores of LG were significantly lower than those of RG (swallowing, p = 0.037; trouble with social eating, p = 0.025). Other scores did not indicate significant differences between LG and RG (pain, p = 0.731; sensory problems, p = 0.152; speech problems, p = 0.112; trouble with social contact, p = 0.142; less sexuality, p = 0.082; teeth, p = 0.349; mouth opening, p = 0.412; dry mouth, p = 0.139; sticky saliva, p = 0.100; coughing, p = 0.643; feeling ill, p = 0.359; pain killers, p = 0.762; nutritional supplements, p = 0.083; feeding tube, p = 1.000; weight loss, p = 0.412; weight gain, p = 0.123).

At 6M, there was no significant difference between LG and RG (pain, p = 0.540; swallowing, p = 0.703; senses problems, p = 0.977; speech problems, p = 0.775; trouble with social eating, p = 0.176; trouble with social contact, p = 0.934; less sexuality, p = 0.137; teeth, p = 0.288; mouth opening, p = 0.181; dry mouth, p = 0.567; sticky saliva, p = 0.265; coughing, p = 0.171; feeling ill, p = 0.057; pain killers, p = 0.329; nutritional supplements, p = 0.301; feeding tube, p = 1.000; weight loss, p = 0.808; weight gain, p = 0.223).

## Discussion

The results of this study indicated that there were no significant differences in any functional measurements between LG and RG at any assessment period. In contrast, the QOL assessments indicated no significant difference till 1M; however, after 3M, some items of QOL assessments showed significantly lower scores in RG compared with LG.

In this study, MTP was measured using a balloon at the tip of a probe placed behind the upper front teeth. This indicates that the balloon was located near the midline of the palate. A previous study reported that a small TC surgical area has little effect on tongue function [17,18]. In this study, 34 (85%) participants underwent partial glossectomy, which means that most participants had limited tongue movement. Thus, there were no significant differences in the MTP or feeding function between the groups. The participants in this study underwent rehabilitation after surgical treatment. Tongue rehabilitation, such as motor training, has been reported to improve tongue pressure in patients undergoing TC treatment [9]. This could help improve the participants’ MTP. Moreover, a previous study reported that a small area of tongue resection did not affect the LCF [9]. Similarly, the participants in this study underwent a small surgical treatment for TC, such as partial glossectomy or hemiglossectomy, indicating no effect on LCF. Thus, there were no significant differences between the groups.

Based on QOL measurements, there was no significant difference between LG and RG at baseline and 1M; however, there were significant differences at 3M and 6M.

At baseline, the participants were not affected by scarring resulting from the surgical treatment for TC. However, some of the participants likely experienced cancer pain. Cancer pain has a significant effect on patients’ QOL. Both EORTC QLQ-C30 and H&N35 include pain-related assessment (EORTC QLQ C30, pain; H&N35, pain killer), and it has been reported that cancer pain is noted more frequently in advanced cancer than in early-stage cancer [19]. It is likely that the participants included in this study had early-stage TC (T1 or T2) and that their cancer pain was localized. Therefore, the effect on cancer pain was considered small with or without the use of painkillers. One possible reason for the lack of a significant difference between LG and RG might be the effects of the surgical treatment for TC. Generally, it takes more than one month for wound healing to be completed after surgical treatment for TC [20]. Thus, the participants were presumably aware of pain and postoperative dysfunction, regardless of the surgical area.

The results of this study indicated a significant difference between LG and RG in QOL measurements at 3M and 6M; however, there were no significant differences in functional measurements. These results suggest that participants who received right-sided TC treatment still experienced discomfort, such as difficulty in swallowing, compared to participants who received left-sided TC treatment. No previous study has investigated the left-right difference in tongue function during swallowing. Regarding mastication, it has been reported that there is a left-right difference called habitual mastication [21,22], and left-right differences in tongue function have been reported [12]. Both the dominant hand and habitual masticatory side have been reported to be more common on the right side [21,23], and the same might be true for tongue function. Therefore, the function of the tongue may not be the same on the left and right sides, and there may be a dominant side. This might be one of the reasons why RG had a lower QOL score than LG, although this was not noted in the functional measurements. However, this study did not investigate the participants’ dominant hand and habitual masticatory side, and it is necessary to investigate the relationship between these factors.

A previous study reported that approximately 30% of patients with early-stage head and neck cancer did not return to their QOL, especially global health status, 12 months after treatment, although their oral function returned before treatment [6]. In this study, we did not compare the changes in QOL measurements over time. However, LG tended to have a higher global health status score at 6M than at BL. On the other hand, RG tended to have a lower global health status score at 6M than at BL. One of the possible reasons for only the global health status indicating a significant difference at 6M might be that the global health status reflected the results of multiple questions compared to other items. The participants did not show a significant functional decline at 6M, and it might be possible that this was not a significant change in subjective symptoms. It is necessary to compare the details of these changes over time in future studies.

Limitations

This prospective cohort study included a small sample size with a further reduction in cases due to patient withdrawal during the study. Patient dropout during a prospective head and neck cancer study due to cancer recurrence and change of residence is often noted [24,25]. Furthermore, we did not assess the patients’ speech function. Moreover, we investigated only the left-right difference and did not investigate the anteroposterior position. It has been reported that the anteroposterior position of the tongue had differences in speech and eating function [26,27]. Therefore, it is necessary to assess the speech function and differences in the anteroposterior area of the tongue in future studies.

## Conclusions

No functional impairment of the tongue was observed on the left and right sides of the surgical site after partial glossectomy or hemiglossectomy. However, the patients’ QOL after treatment was significantly lower on the right side compared with the left side. This suggests that the left-right difference in the primary site should be considered in postoperative care.
